# Canine Parvovirus *ns1* gene and Chicken Anemia *vp3* gene induce partial oncolysis of Canine Transmissible Venereal Tumor

**DOI:** 10.1038/s41598-017-15734-6

**Published:** 2017-11-13

**Authors:** Aubid Hussain Bhat, Bhaskar Ganguly, Ashok Kumar Tiwari, Arup Kumar Das

**Affiliations:** 1 Department of Surgery and Radiology, College of Veterinary & Animal Sciences, G. B. Pant University of Agriculture & Technology, Pantnagar, 263145 India; 2 Animal Biotechnology Center, Department of Veterinary Physiology and Biochemistry, College of Veterinary & Animal Sciences, G. B. Pant University of Agriculture & Technology, Pantnagar, 263145 India; 3 Molecular Biology Laboratory, Division of Veterinary Biotechnology, Indian Veterinary Research Institute, Izzatnagar, 243122 India

## Abstract

The oncolytic effect of Canine Parvovirus *ns1* gene and Chicken Anemia *vp3* gene in naturally occurring cases of Canine Transmissible Venereal Tumor (CTVT) is being reported. Dogs suffering from CTVT (N = 18) were systematically randomized into three groups *viz*. A, B, and C (n = 6). Animals of the groups A, B, and C received 100 µg of the *ns1* gene, *vp3* gene, and *ns1* **+** *vp3* gene combination, respectively, for three weeks intratumorally at weekly intervals; results were normalized against base values before commencement of therapy and after complete remission that were taken as negative and positive controls, respectively. Initiation of oncolytic gene therapy arrested the further progression of the tumor but most of the animals in the study underwent incomplete remission, indicating incomplete activity of *ns1* and *vp3* genes. The oncolytic effect of the treatments was in the order *ns1* > *vp3* > *ns1* + *vp3*. Oncolysis was accompanied by decreased mitotic index and AgNOR count, and increased TUNEL positive cells and CD4^+^ lymphocyte counts. Our findings show that Canine Parvovirus *ns1* may eventually find an important role as an oncolytic agent.

## Introduction

Oncolytic viruses replicate preferentially in cancer cells and kill them at the end of the replication cycle. Live viruses were first used for the treatment of cancer more than a century ago. However, soon after its inception, the approach had to be abandoned due to problems of toxicity, development of antiviral immunity and risk of re-emergence of virulent viruses^1^. Lately, there has been a revival of scientific interest in the development of virus-based cancer therapeutics. Molecular biotechnology has allowed genetic engineering of viruses for enhanced selectivity towards tumor cells – an approach that was first demonstrated with Herpes Simplex virus type-1 (HSV-1) in an experimental glioma model^2^. Successively, several viruses with high oncolytic capacity have been identified as potential candidates for clinical applications. Chicken anemia virus and canine parvovirus are two such viruses that possess inherent oncolytic properties^3^.

The chicken anemia viral protein (VP) 3, also termed apoptin, specifically, kills tumor cells while sparing normal cells. Upon expression in normal cells, apoptin is accumulated in the cytoplasm whereas in cancer cells, it is specifically targeted to the nucleus where it elicits its lethal effects^4^. Canine parvovirus-2 (CPV-2) is an extremely simple DNA virus that encodes one to two early products and a limited number of late structural proteins. It lacks mechanisms for inducing S phase and replicates only in proliferating host cells. This feature confers additional selectivity for killing rapidly growing cancer cells. The apoptosis-inducing activity of parvoviruses was mapped to the non-structural protein-1,-2 (*ns*1/*ns*2) of minute virus of mice (MVM) and *ns*1 of parvovirus B19^5^. Unlike apoptin, *ns1* does not rely solely on apoptosis for its oncolytic effect.

Biological similarities with humans make the dog an ideal comparative model system for studying new cancer treatments^6^. Canine transmissible venereal tumor (CTVT) is the most common cancer of dogs in many parts of the world. It is a unique, naturally transmissible, contagious tumor, where the mutated tumor cell itself is the causative agent and perpetuates as a parasitic allograft. Moreover, CTVT is regarded as a model for studying the immune responses that are involved in tumor regression^7^. For a detailed discussion on CTVT, the reader is referred to a comprehensive review of this cancer by Ganguly *et al*.^8^. Few studies have assessed the therapeutic efficacy of *ns1* and *vp3* genes against tumors *in vitro*, and *in vivo* studies are fewer still. We report the comparative oncolytic effects of Canine Parvovirus *ns1* gene and Chicken Anemia *vp3* gene on CTVT. To the best of our knowledge and belief, this is the first study on the use of oncolytic viruses against CTVT.

## Results and Discussion

All cases in the study presented cauliflower-like nodular masses of varying size (0.5–5 cm in diameter) with ulcerated surfaces that are characteristic of TVT. In males, the tumor mostly (five of six) appeared as solitary, small to large, sessile or pedunculated, soft nodular mass at the base of the penis. In females, the lesions were usually solitary, cauliflower-like in the vulvar region. All the tumors were irregular, soft to relatively firm, and fragile. The tumors were of fulminating nature in few (two of eighteen) cases, causing the animals to turn cachectic. Primary genital tumor in the male dog was usually found in the caudal part of the penis, from the crura to the bulbous glans, or the area of the glans penis, and occasionally on the prepuce. In some (two of six) cases, the tumors were very large and extended to the scrotum, causing difficulty in exposing the penis. The neoplastic masses were intense pink to bright red in color and bled spontaneously in both sexes. Persistent or intermittent serosanguinous discharge, often foul smelling, was evident and most of the animals also exhibited constant licking of the genitalia. Only one case suffered retention of urine due to the pressure of the growth on the urinary tract. These findings on the physical appearance and location of the lesions were consistent with previous reports^8^. Extragenital lesions, primary or metaplastic, were not evident in any case.

**Figure 1:**
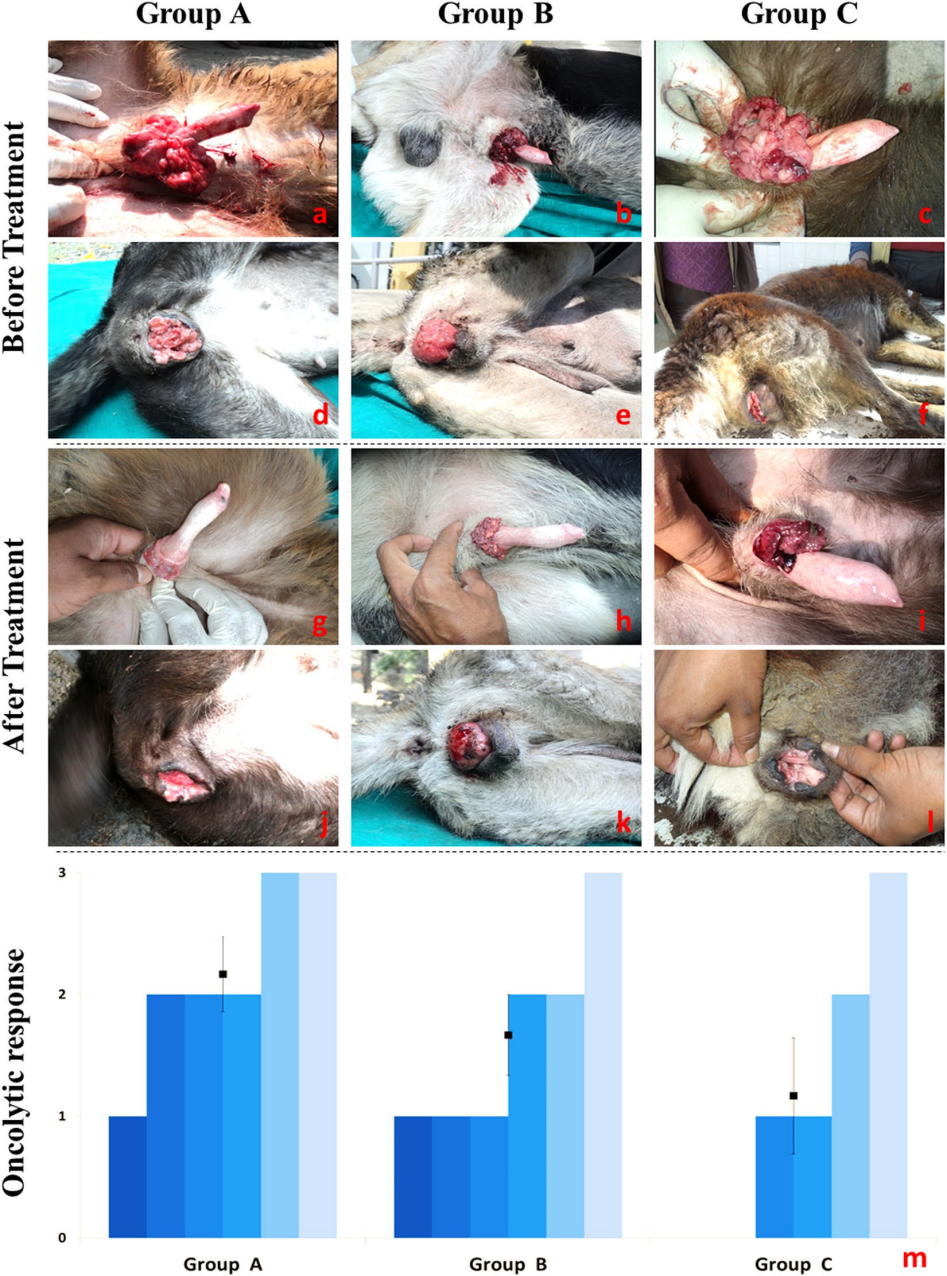
Representative photographs of dogs before initiation (males, (**a**–**c**); females, (**d**–**f**) and after completion (males, (**g–i**); females, (**j**–**l**) of therapy. Group-wise individual and mean (±SE) oncolytic response (**m**) have been shown for comparison.

Representative photographs showing the gross appearance of the lesions before and after treatment with oncolytic genes are shown for comparison in Fig. 1. In most of the animals, signs of mild to moderate tumor regression such as tumor contraction, blackening, ulceration, and a decrease in tumor size were observed, revealing the incomplete oncolytic activity of *ns1* and *vp3* genes. Any untoward effects of oncolytic gene therapy were not evident in any of the groups. However, it was observed that tumor regression was stunted between 10 to 14 days of gene therapy. In all cases, including those where complete regression did not occur, initiation of oncolytic gene therapy arrested the further progression of the tumor. Rapidly progressing cancers, where the immune functions remain intact, may thus benefit most from similar treatments; the oncolytic gene therapy can act to contain the progression of cancer and the immune system can cleanse out the existing tumorous cells. The greatest oncolytic effect was seen in Group A although the differences between the groups were statistically non-significant (*p* > 0.1).

**Figure 2:**
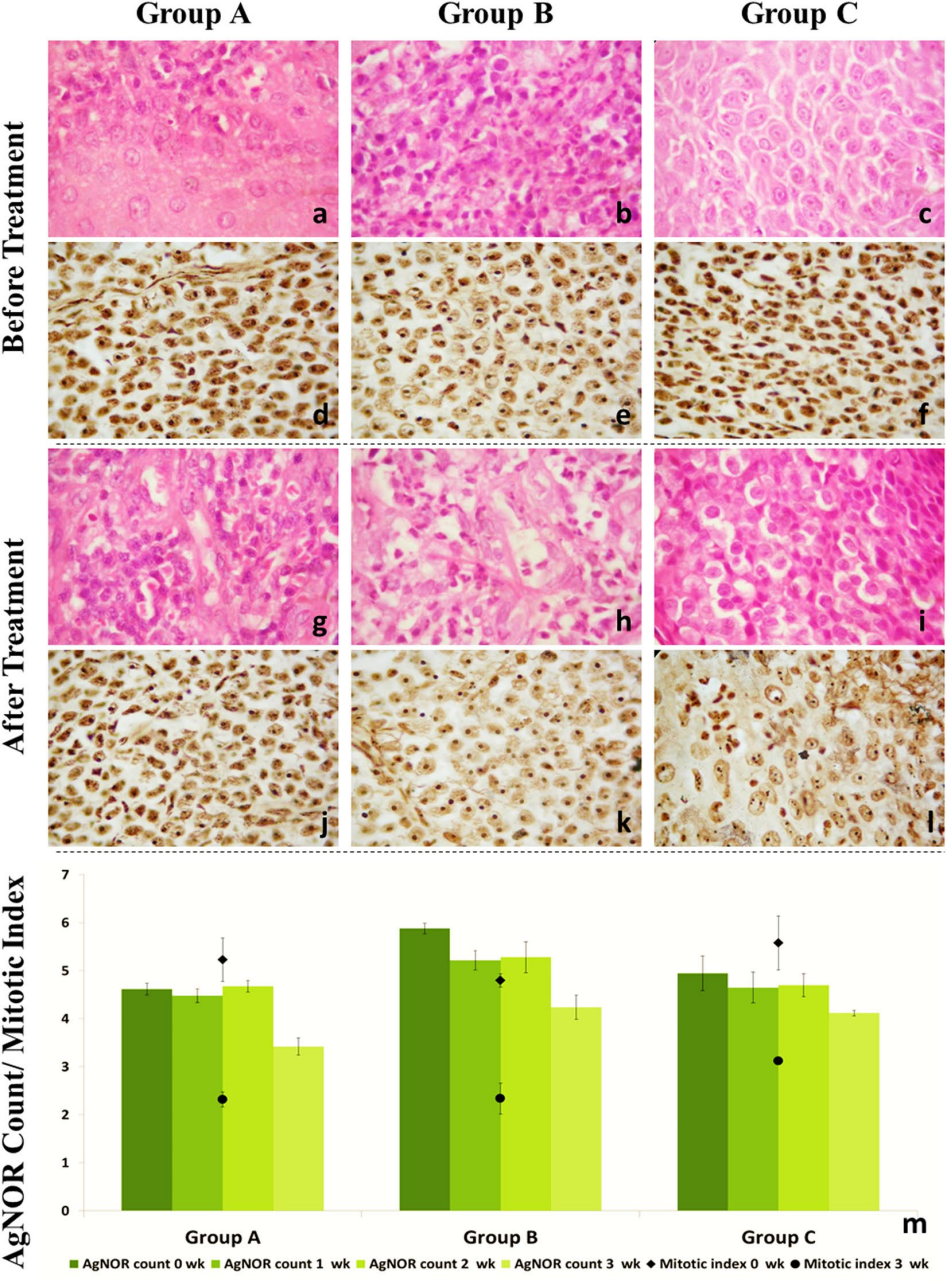
Representative photomicrographs (1000×) showing histopathological appearance and AgNOR staining of tissue sections obtained before initiation (H&E, (**a**–**c**); AgNOR count, (**d**–**f**) and after completion (H&E, (**g–i**); AgNOR count, (**j**–**l**) of therapy. Group-wise mean (±SE) AgNOR counts and mitotic indices (**m**) have been shown for comparison.

Histological examination (Fig. 2) of neoplastic tissue on day zero showed high mitotic index, pleomorphic cells, proliferating fibroblasts, neoplastic cells with high nuclear: cytoplasmic ratio, low infiltration of mononuclear cells and the presence of a moderate network of blood vessels associated with stromal tissue neovascularization. The TVT cells frequently showed apoptotic characteristics, such as shrunk anoikic cells with condensed chromatin and fragmented nuclei. The oval to round neoplastic cells often appeared in sheet-like confluent clusters. Multifocal hemorrhages were also noticed in few sections. The cells were closely packed together with strands of fibrous connective tissue. The nuclei were centrally placed, large in size, round to oval in shape, granular and vesicular. The cytoplasm was faintly basophilic, vacuolated and moderate in amount. Under high magnification, the prominent nuclei frequently presented mitotic figures, signifying the proliferating nature of the tumor. The few infiltrating mononuclear cells were scattered throughout the tumor parenchyma. Similar features of TVT have been reported consistently^8,9^.

Within three weeks of therapy, both *ns1* and *vp3* primarily affected the nuclei of the neoplastic cells causing condensation, karyorrhexis, and karyolysis leading to decreased tumor cell numbers, a corresponding increase in the fibrous tissue and marked infiltration of lymphoid cells. On day 21 post-treatment, infiltration of mononuclear and polymorphonuclear cells, the formation of cystic spaces, an increase in the extracellular matrix, and a decrease in proliferating cells was observed in *ns1* or *vp3* treated groups. The histopathological sections from the animals of Group A showed fewer neoplastic cells, initiation of regression, and coarse and granular nuclei with scanty vacuolation of cytoplasm. The magnitude of lymphocyte infiltration was greater in the animals of Group A than in the animals of Groups B and C. There was an almost complete regression of tumor by day 28 following *ns1* gene therapy. In Group C, no apparent changes were seen in the microscopic appearance.

A significant (*p* < 0.05) decrease in AgNOR index was observed in Groups A and B, whereas the decrease was non-significant in the animals of Group C. There were no significant differences in the AgNOR indices at zero (4.62 ± 0.12 *vs*. 5.88 ± 0.11), first (4.48 ± 0.14 *vs*. 5.22 ± 0.20) and second week (4.68 ± 0.12 *vs*. 5.28 ± 0.32) of the animals of Groups A and B, respectively. In Group C, the AgNOR indices at zero (4.95 ± 0.36) and first week (4.65 ± 0.32) differed non-significantly with the value at third week (4.12 ± 0.06). The lowest AgNOR count was recorded at third week (3.42 ± 0.18) in Group A. Group-wise representative histopathological sections, AgNOR counts and mitotic indices have been shown in Fig. 2 for comparison.

The significantly higher initial AgNOR index reflects the aggressiveness of TVT and of associated chromosomal disorders; high initial AgNOR counts have been associated with a high degree of cell proliferation and active mitotic stage^10–13^. A significant decrease in AgNOR indices in the animals of Groups A and B at third week in comparison to their base value at zero weeks may be indicative of tumor regression stages. It has been demonstrated that malignant cells have more AgNOR protein as compared to non-malignant cells^13,14^. Moreover, in cancer tissues, AgNOR protein expression was found to be strictly related to the cell duplication rate and variation in AgNOR size and dispersion was higher in malignant than in benign neoplasms^11^.

**Figure 3:**
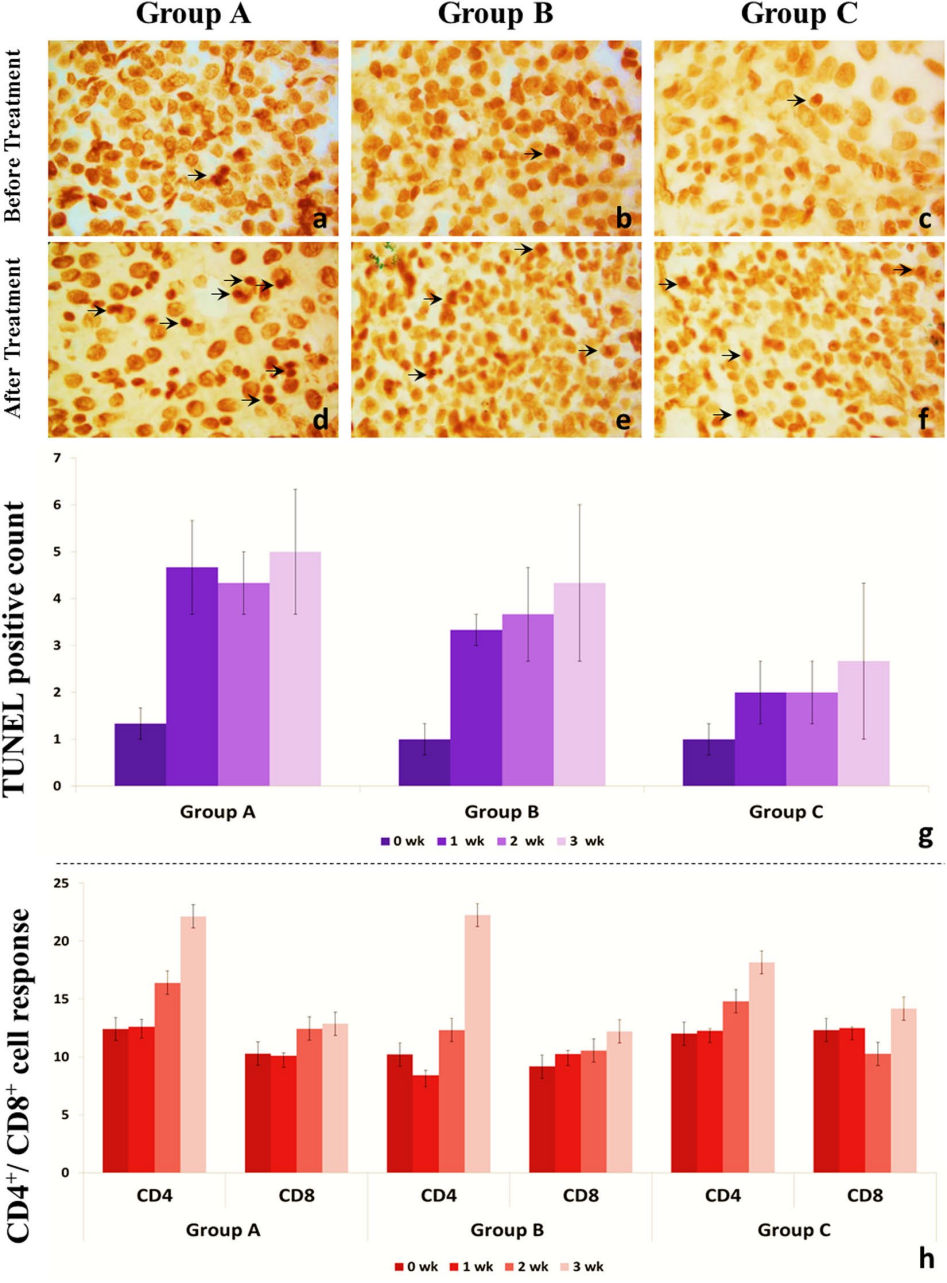
Representative photomicrographs (1000×) showing TUNEL positive cell counts in tissue sections obtained before initiation (**a**–**c**) and after completion (**d**–**f**) of therapy. Group-wise mean (±SE) TUNEL positive cell counts per field (**g**) have been shown for comparison. Group-wise mean (±SE) percentage of circulating CD4^+^ and CD8^+^ cell counts (**h**) have been shown for comparison.

The number of TUNEL positive nuclei (Fig. 3a–g) was higher in the *ns1* and *vp3* treated groups as compared to the base values. Few TUNEL positive cells were found at zero weeks in the Groups A, B, and C and their number increased significantly in the animals of Groups A and B with the initiation of therapy. TUNEL positive cells are more abundant during the regression phase than in the growing phase of TVT^15^. Hill *et al*.^16^ and Cockrill and Beasly^17^ have reported that the regression of TVT is due to the necrosis of neoplastic cells; findings on TUNEL positivity in the present study support the involvement of apoptosis, at least following gene therapy with *ns1* and *vp3* genes, in the regression of TVT.

Excepting the increase in the number of CD4^+^ lymphocytes in Groups A and B in the third week, the number of T-lymphocyte subtypes CD4^+^ and CD8^+^ did not vary considerably during most of the study period (Fig. 3h). This contradicts extant literature on the subject where increased proportions of these cells have been described during the regression phase. Gonzalez *et al*.^18^. demonstrated that the number of CD4^+^ and CD8^+^ lymphocytes were significantly greater during the regression phase of TVT (52.4%) as compared to its progression phase (39.6%); in both phases, the number of CD8^+^ cells was greater than the number of CD4^+^ cells.

Comparing the findings with the study of Hsiao *et al*.^19^, it can be suggested that the lymphocytes that were seen in large quantities during the progression phase of the present experiment did not impede tumor growth. This emphasizes that factors within the tumor environment may have been responsible for the incompetence of these lymphocytes. TVT cells secrete several toxins and factors, TGF-β1, for example, which cause apoptosis of B-lymphocytes and alter the proportions of CD4^+^ and CD8^+^ lymphocytes^8^. It has been hypothesized that oncolytic viruses can cause tumor cells to reduce immunomodulating factors^20^. The high levels of CD4^+^ cells during the third week in Groups A and B, which showed the greatest oncolytic response, suggests a role of this lymphocyte subtype in TVT regression following *ns1* and *vp3* gene therapy. Chuang *et al*.^21^ observed a dramatic increase in lymphocyte infiltration of the tumor mass after *IL-12* gene therapy and CD8^+^ lymphocytes formed the major subtype in these tumor-infiltrating lymphocytes (TILs). Our findings do not support a predominant role of CD8^+^ cells during such regression processes. Although CD8^+^ cytotoxic T-lymphocytes (CTLs) play important role in tumor regression, CD4^+^ cells are necessary for entry of CTLs in the tumor tissue^22^. TVT cells completely lack MHC II expression^8^. The accentuated CD4^+^ helper T-cell response may be a compensatory mechanism to compensate the newly-regained, low level of MHC II expression during the regression phase. Further, the stimulation of a particular lymphocyte subtype may depend on the oncolytic or therapeutic agent being used. Much lower CD4^+^ and CD8^+^ counts have been reported during spontaneous regression of untreated, experimentally-induced TVT cases^23^.

In this study, we could demonstrate partial oncolysis of CTVT by three weekly intratumoral injections of 100 μg of either canine parvovirus *ns1* gene, chicken anemia *vp3* gene or a combination of *ns1* gene and *vp3* gene; the oncolytic effect achieved was in the order *ns1* > *vp3* > *ns1* + *vp3*. The extent of oncolysis achieved with viral gene therapy in the present study is not convincing enough for discontinuation of conventional chemotherapy of TVT with vincristine that offers near-absolute success. Also, the effect of viral gene construct injected intratumorally at one site on metastatic lesions, when present, remains questionable. Oncolysis was accompanied by a decrease in mitotic index and AgNOR count, and an increase in TUNEL positive cells and circulating CD4^+^ lymphocyte counts, indicating the involvement of apoptosis and helper T-cell activity in the regression process. While further evidence is needed to show if the regression of TVT is due solely to the direct oncolytic activity of *ns1* and *vp3* or to the indirect activation of apoptotic and helper T-cell activity or a combination of both, the study shows that the viral gene therapy, especially with *ns1*, may eventually play an important role as an oncolytic agent. The greatest benefit of such therapies is likely to be realized in cancers where the immune system remains otherwise functional.

## Methods

### Experimental design

Eighteen adult dogs (N = 18; 6 male and 12 female dogs), visiting the Teaching Veterinary Clinical Complex, Pantnagar, affected with naturally occurring CTVT and clinically free from any other disease were divided into three groups (*viz*. A, B, and C) using systematic random allocation proportionate to sample sex ratio (*i.e*. n = 6; 2 male and 4 female dogs per group). Animals of the groups A, B, and C received 100 µg of the *ns1* gene, *vp3* gene, and *ns1* **+** *vp3* gene combination, respectively, for three weeks, intratumorally, at weekly intervals. The inability of the vector, which lacks the oncolytic inserts, to induce oncolysis has been established previously in a cell-line^3^ and a rat model^24^. In a clinical setting, it was not ethically acceptable to allocate patients to a treatment that was known to have no apparent benefit; hence, the base values of the TVT patients before the commencement of therapy and after complete remission were taken as negative and positive controls, respectively. All cases undergoing incomplete remission were treated with standard vincristine therapy^8^.

### Gene constructs

The constructs pVIVO.vp3, pVIVO.ns1, and pVIVO.vp3.ns1 were gratuitously provided by the Division of Veterinary Biotechnology, Indian Veterinary Research Institute. For details of the preparation, characterization, expression potential of the constructs and their *in vitro* oncolytic effects, the readers are referred to Saxena *et al*.^3^ and Santra *et al*.^24,25^.

### Assessment of oncolysis

The regression of the tumor mass was graded independently by three veterinarians as nil, mild (up to 25%), moderate (up to 50%) or complete (up to 100%), and the response to oncolytic therapy was scored as 0, 1, 2 or 3, respectively.

### Histological examination and Determination of Mitotic index

Histological examination was performed in five-micrometer thick sections stained with hematoxylin and eosin^26^, and lesions were recorded microscopically. Mitotic index was determined as described by Yu *et al*.^27^.

### AgNOR staining

Argyrophilic nuclear organizing regions (NORs) were stained as described previously^28^. The uptake of this stain correlates with the rapidity of proliferation of the tumor cells. NORs appeared as dark spots within the orange colored nuclear background. Clustered spots with indistinguishable boundaries were counted as a single spot.

### Terminal Deoxynucleotidyl Transferase-mediated dUTP Nick End Labeling (TUNEL)

TUNEL assay was performed in paraffin-embedded tissue sections using DeadEnd colorimetric TUNEL system (Promega). Briefly, tissue sections were dewaxed, hydrated, fixed with 4% paraformaldehyde, and permeabilized with proteinase-K at room temperature. Thereafter, the sections were left covered with 25 µM biotinylated nucleotide and 25 U of terminal deoxynucleotidyl transferase for 1 h at 37 °C. The reaction was stopped with 2× saline sodium citrate, and the endogenous peroxidase was blocked with 0.3% hydrogen peroxide for 4 min at room temperature. Fragmentation of DNA was detected on the basis of binding of streptavidin-horseradish peroxidase to the biotinylated nucleotides and visualizing the brown color following incubation with hydrogen peroxide and diaminobenzidine. TUNEL positive cells showed dark brown apoptotic nuclei.

### CD4^+^/CD8^+^ response

Changes in the number of circulating CD4^+^ and CD8^+^ cells were estimated by direct staining with phycoerythrin-conjugated monoclonal antibodies (Dog T-Lymphocyte Cocktail, BD Biosciences) and enumeration in a FACScan flow cytometer (Becton Dickinson).

### Statistical Analysis

Statistical inferences were drawn on the basis of one-way analysis of variance (ANOVA). Critical difference test was used to determine the significance of the difference between two means.

### Data availability statement

All relevant data pertaining to the study shall be made available upon request.

### Approval

All sampling and experimental procedures involving animals were in accordance with Breeding of and Experiments on Animals (Control and Supervision) Amendment Rules, Government of India, 2001, and were approved by the Institutional Animal Ethics Committee of the College of Veterinary and Animal Sciences, G. B. Pant University of Agriculture and Technology, Pantnagar *vide* Approval No. IAEC/VSR/CVASc/114, dated 09.11.2012.
